# Effects of *NRAS* Mutations on Leukemogenesis and Targeting of Children With Acute Lymphoblastic Leukemia

**DOI:** 10.3389/fcell.2022.712484

**Published:** 2022-02-08

**Authors:** Jiabi Qian, Zifeng Li, Kunlin Pei, Ziping Li, Chunjie Li, Muxia Yan, Maoxiang Qian, Yuanbin Song, Hui Zhang, Yingyi He

**Affiliations:** ^1^ Guangzhou Women and Children’s Medical Center, Institute of Pediatrics, Guangzhou, China; ^2^ Department of Hematology/Oncology, Guangzhou Women and Children’s Medical Center, Guangzhou, China; ^3^ Department of Hematology and Oncology, The Shanghai Key Laboratory of Medical Epigenetics, International Co-laboratory of Medical Epigenetics and Metabolism, Institute of Pediatrics, Institutes of Biomedical Sciences, Children’s Hospital of Fudan University, Ministry of Science and Technology, Fudan University, Shanghai, China; ^4^ Department of Hematology and Oncology, National Children’s Medical Center, Children’s Hospital of Fudan University, Shanghai, China; ^5^ Department of Hematologic Oncology, State Key Laboratory of Oncology in South China, Collaborative Innovation Center for Cancer Medicine, Sun Yat-sen University Cancer Center, Guangzhou, China

**Keywords:** NRAS proto-oncogene, acute lymphoblastic leukemia, signaling pathway activation, therapeutic targeting, leukemogenic potential

## Abstract

Through the advancements in recent decades, childhood acute lymphoblastic leukemia (ALL) is gradually becoming a highly curable disease. However, the truth is there remaining relapse in ∼15% of ALL cases with dismal outcomes. *RAS* mutations, in particular *NRAS* mutations, were predominant mutations affecting relapse susceptibility. *KRAS* mutations targeting has been successfully exploited, while NRAS mutation targeting remains to be explored due to its complicated and compensatory mechanisms. Using targeted sequencing, we profiled *RAS* mutations in 333 primary and 18 relapsed ALL patients and examined their impact on ALL leukemogenesis, therapeutic potential, and treatment outcome. Cumulative analysis showed that *RAS* mutations were associated with a higher relapse incidence in children with ALL. *In vitro* cellular assays revealed that about one-third of the *NRAS* mutations significantly transformed Ba/F3 cells as measured by IL3-independent growth. Meanwhile, we applied a high-throughput drug screening method to characterize variable mutation-related candidate targeted agents and uncovered that leukemogenic-*NRAS* mutations might respond to MEK, autophagy, Akt, EGFR signaling, Polo−like Kinase, Src signaling, and TGF−*β* receptor inhibition depending on the mutation profile.

## Introduction

Translational genomic research and risk stratification-directed therapy have gradually made childhood acute lymphoblastic leukemia (ALL) a highly curable cancers (Vora et al., 2013; Pui et al., 2018), with over 90% leukemia-free survival in developed countries. However, about 15–20% children with ALL eventually relapse with dismal outcome (Mullighan et al., 2008; Ding et al., 2012; Bhojwani and Pui, 2013; Meyer et al., 2013; Pierro et al., 2017; Brown and Ferrando, 2018). Among the genetic alterations, *RAS* mutations, in particular *NRAS* mutations, are over presented in children with ALL (Ma et al., 2015). Studies have shown that the prevalence of *NRAS* mutations varies from 15 to 34% in children with ALL (Case et al., 2008; Irving et al., 2014; Ma et al., 2015). Impressively, Ma et al. has reported that *NRAS* mutations conferred susceptibilities on B cell ALL (B-ALL) relapse (Ma et al., 2015). Consequently, the oncogenic mutations in the NRAS represented crucial therapeutic targets (Ward et al., 2012). Therefore, it’s highly needed to explore the translational potential of NRAS mutations in pediatric ALL.

RAS GTPase (HRAS, KRAS and NRAS) family members play a critical role in human malignancies via regulating cell growth, differentiation, survival, motility, and adhesion through transmitting signals to activate downstream signaling cascades, including the RAF-MEK-ERK and PI3K-AKT pathways (Karnoub and Weinberg, 2008; Stephen et al., 2014; Burgess et al., 2017). In this regard, NRAS mutations have been found to be able to lead to constitutive activation, which in turn activate its downstream signaling pathways, including mitogen-activated protein kinase (MAPK), phosphatidylinositol 3-kinase (PI3K)-AKT, and others (i.e., RalGDS, and janus kinase (JAK) - signal transducer and activator of transcription (STAT)) (Brunet et al., 1999; Cox and Der, 2003; Downward, 2003; Xu et al., 2007; Wang et al., 2013; Kong et al., 2014; Zhang and Cheong, 2016; Bery et al., 2018). In the therapeutic targeting facet, much attentions have been paid to the breakthrough of KRAS^G12C^ targeting by several small molecules, such as AMG-510, MRTX849, and ARS-1620 (Janes et al., 2018; Canon et al., 2019; Hallin et al., 2020). Moreover, the KRAS^G12C^ targeting has been successfully translated into clinics with very promising results (Lito et al., 2016; Hallin et al., 2020). However, effective NRAS targeting remains to be explored.

It’s well established that NRAS stimulates proliferation through activating RAS-RAF-MAPK-ERK signaling pathway. Unfortunately, trials using ERK or MEK inhibitors to treated leukemic patients with NRAS mutations do not generate satisfactory results as expected. For example, Jain et al. has reported that three AML patients with *NRAS* mutations fail to respond to the MAPK inhibitor (selumetinib [AZD6244]) (Jain et al., 2014). Similarly, the reported NRAS-targeting agents have failed to demonstrate the satisfying outcomes. Furthermore, multiple *in vitro* and *in vivo* evidences has shown that *NRAS* mutated myeloma and/or leukemic cells are resistant to KRAS^G12C^-targeted small molecules (Welsch et al., 2017; Janes et al., 2018; Canon et al., 2019; Hallin et al., 2020), indicating the specificity of NRAS targeting. Taken together, all these evidence has pointed out that the complex NRAS downstream signals and their compensatory effect might be the bottle-neck of precise targeting (Posch et al., 2013; Samatar and Poulikakos, 2014).

To this end, we retrospectively evaluated the impact of *RAS* mutations on children with ALL enrolled onto CCCG-ALL-2015 clinical trial and tested the contributions of *NRAS* mutations on ALL leukemogenesis and drug response. Furthermore, we utilized high-throughput drug screening (HDS) method to explore the candidates for *NRAS* targeting.

## Methods

### Patients

Newly Diagnosed (*N* = 333) and relapsed (*N* = 18) B-ALL patients enrolled onto CCCG-ALL-2015 clinical trial were included for this study. Ethical approval was obtained from the ethics committee at Guangzhou Women and Children’s Medical Centre 2015020936, 2017102307, and 2020-04500). Informed consent was provided by the patients’ legal guardians, or patients themselves if they were over 8 years old according to the Helsinki Declaration, and their related clinical information was collected for this study. The survival and relapse analyses were performed using Cox proportional hazards regression model.

### Reagents and Cell Lines

All the reagents used in this study were listed in the Supplementary Table S1. The HEK-293T cells were purchased from the American Type Culture Collection (ATCC, United States), and Ba/F3 cells were gifted by Jun Yang at St. Jude Children’s Research Hospital (Xu et al., 2015). The HEK-293T cells were maintained in Dulbecco’s Modified Eagle Medium (DMEM) (Invitrogen, United Kingdom) supplemented with 10% fetal bovine serum, and the Ba/F3 cells were maintained in RPMI1640 supplemented with 10% fetal bovine serum and 10 ng/ml recombinant mouse interleukin 3 (IL3) (PeproTech EC, London, United Kingdom).

### Targeted Next-Generation Sequencing and Validation

DNA was extracted among the diagnostic bone marrow and their matched saliva samples by Trizol (Thermofisher, United States) according to the manufacturer’s protocol. Targeted sequencing of hematological malignancies related genes (Supplementary Table S2) was completed at Kindstar Global (Beijing) Technology, Inc. As detailed, targeted gene capture and library construction for NGS were performed using NimbleGen Sequence Capture Arrays (Roche, Basel, Switzerland) according to the manufacturer’s protocol. Then, the NGS libraries were sent to generate 150-bp paired-end reads for sequencing on the Illumina HiSeq X10 platform (San Diego, CA, United States). Sequencing reads were aligned to the human reference genome (hg19) using Burrows–Wheeler Aligner (BWA-0.7.10). Duplicated reads were then marked and removed using Picard (picard-tools-2.17.0). Variant calls were performed using VarDictJava (1.5.8) (Lai et al., 2016) with pre-curated blacklist variant filters and custom Annovar scripts. Finally, the confident variants were then annotated and manually checked using IGV. Structural variants were called using Delly (Rausch et al., 2012; Hunger and Mullighan, 2015) and filtered using BreakTrans. In the meanwhile, we have retrieved and analyzed the RAS family mutation data from St. Jude PeCan Data Portal (McLeod et al., 2021).

### Cytokine-independent Growth Assay in Ba/F3 Cells

The full-length *NRAS* cDNA was amplified and cloned into the cL20c-IRES-GFP lentiviral vector. *NRAS* mutations were generated using Q5 Site-Directed Mutagenesis Kit (New England Biolabs, United States) with primers listed in Supplementary Table S3. Lentiviral supernatants expressing *NRAS* mutants were generated by transient transfection of HEK-293T cells using Lipofectamine 3000 (Invitrogen, United Kingdom) following the manufacturer’s protocol. Ba/F3 cells were transduced with lentiviral supernatants expressing different NRAS mutants with MOI = 10, following with NRAS expressing cell sorting 48 h after lentiviral transduction by FACSAria II (BD, United States). Then, sorted Ba/F3 cells were washed three times with pre-cold PBS, seeded in the 96-well plate with 1×10^6^/ml cell density, and maintained with full RPMI1640 media in the absence of murine IL3 cytokines. Cell viability was evaluated with Trypan blue using a TC10 automated cell counter (BIO-RAD) daily for at least 7 days.

### High-Throughput Drug Screening Assay

High-throughput drug screening (HDS) was used to evaluate the cytotoxic effect of different candidate agents on NRAS^G12^-transformed Ba/F3 cells (Supplementary Figure S1). Transformed Ba/F3 cells were grown in RPMI160 supplemented with 10% FBS and seeded in a 384-well plate (Corning, NY, United States) at a density of 1200 cells per well. The initial concentration of targeting drugs (Supplementary Table S4) was 10 μM and then serial diluted to generate the drug concentration series (10, 3.3, 1.1, 0.37, 0.12, 0.04, 0.013, 0.0045, 0.0015, and 0.0005 μM). The serial drug concentrations were added to the cells using an automated liquid handling system (PerkinElmer, MA, United States). Cell viability was assessed using CellTiter-Glo™ kits (Promega, WI, United States) after 72 h of drug exposure. The inhibition rate of each drug concentration was calculated after normalization using the formula below. The IC50 was calculated using GraphPad Prism v7.0 (GraphPad Software, Inc.). The HDS experiments were performed in triplicate and independently repeated three times.

$$\text{Inhibition rate}\;\left(\%\right)=100\%-\frac{RLUDrug-RLUBackground}{RLUDMSO-RLUBackground}\times 100\%$$

### Cell Counting Kit-8 (CCK-8) Assays

NRAS^G12^-transformed Ba/F3 cells were seeded at a density of 2×10^5^/ml in a 96-well plate, and treated with increasing doses of tested agents listed in Supplementary Table S1 for 72 h. The cell viability was tested using CCK-8 assay kit (Dojindo Molecular Technologies Inc., Japan) and colorimetric density was measured using a Multiscan MS spectrophotometer (Labsystems, Stockholm, Sweden). The experiments were performed in triplicate and repeated at least three times.

### Western Blotting Assay

Ba/F3 cells with *NRAS* mutants were lysed in 1× lysis buffer (Cell Signaling Technology, United Kingdom). Proteins (20 mg) were electrophoresis on 10% PAGE gel (BIO-RAD) and then transferred onto PVDF membranes. After blocking membranes with 5% milk for 1 h at room temperature, the membranes were incubated with anti- Phospho- Erk1/2 antibody (Cell Signaling Technology, United Kingdom, 4370S, 1:1,000 dilution), anti- Erk1/2 antibody [Cell Signaling Technology, United Kingdom, 4696S, 1:1,000 dilution], anti- Phospho- Stat5 (Tyr694) antibody [Cell Signaling Technology, United Kingdom, 4322S, 1:1,000 dilution], and anti- Stat5 antibody (Cell Signaling Technology, United Kingdom, 94205S, 1:1,000 dilution). Tubulin was used as internal control. The blots were incubated with HRP-conjugated secondary antibodies for 1 h and visualized using the ECL system. All the antibodies we used were listed in Supplementary Table S1.

### Statistical Analysis

All statistical analyses were performed using R (version 3.3.1) and GraphPad Prism v7.0 (GraphPad Software, Inc.). Kaplan–Meier survival analysis was performed and survival differences between groups were assessed with the log-rank test, assuming significance at *p* < 0.05. The other data values were presented as the mean ± SD. Statistical analysis methods were denoted in independent figure legends. *p* < 0.05 was considered statistically significant.

## Results

### RAS Family Alterations in Acute Lymphoblastic Leukemia Patients

**FIGURE 1:**
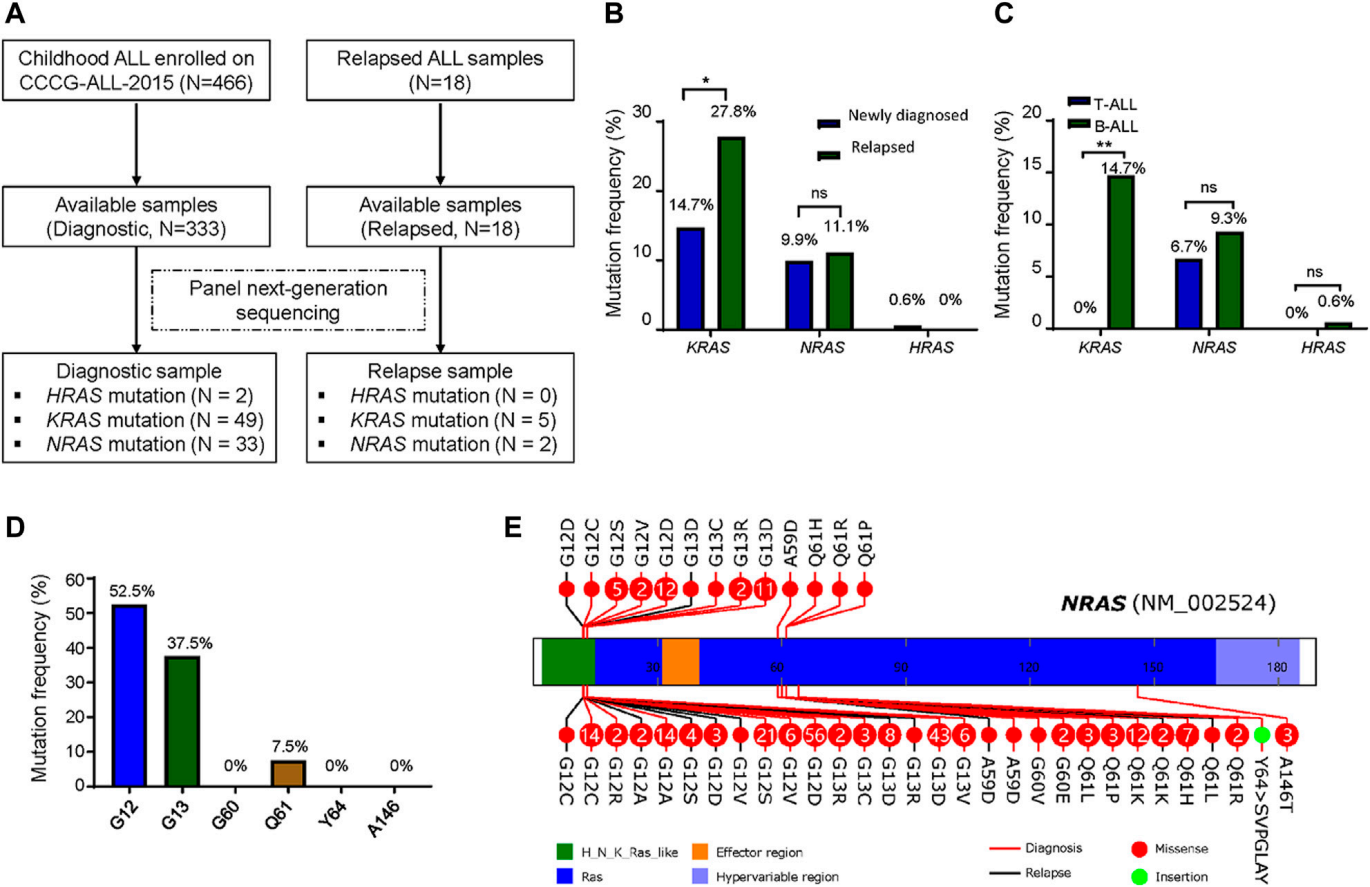
*RAS* mutations in patients with newly diagnosed and relapsed ALL. **(A)** Identification of *HRAS*, *KRAS* and *NRAS* mutations in patients with newly diagnosed and relapsed ALL enrolled onto CCCG-ALL-2015 clinical trial at Guangzhou Women and Children’s Medical Center. **(B)** *RAS* mutations frequency between newly diagnosed (*N* = 333) and relapsed (*N* = 18) cases. **(C)** *RAS* mutations frequency between diagnostic T-ALL and B-ALL. **(D)** *NRAS* mutations frequency including G12, G13, G60, Q61, Y64 and A146 residues in this study cohort. **(E)** *NRAS* mutation profile from our study cohort (upper panel) and PCGP study cohort. *NRAS* mutations spanned the full length of the gene (upper panel, our study cohort; lower panel, PCGP; red line, newly diagnosed ALL; black line, relapsed ALL; solid red circle, missense mutations; solid green circle, insertion mutations; number in this circle represents case number). McNemar chi-square test was performed to compare the frequency of *KRAS*, *NRAS*, and *HRAS*. *p* < 0.05 (*, <0.05; **, <0.01) was considered statistically significant.

Total 333 children with newly diagnosed ALL and 18 children with relapsed ALL from CCCG-ALL-2015 study at the Guangzhou Women and Children’s Medical Center were enrolled onto this study (Table 1; Figure 1A; Supplementary Figure S2). Targeted next-generation sequencing was performed to identify the ALL-related genetic alterations. We first analyzed the RAS mutation frequency and profile among newly diagnosed patients. As shown in Figure 1B, the frequency of *KRAS*, *NRAS*, and *HRAS* was 14.7, 9.9, and 0.6% respectively, while the frequency of *K-*, *N-*, and *H- RAS* mutation among relapsed patients was 27.8, 11.1, and 0% respectively (Figure 1B; Supplementary Tables S5–S7). Notably, *KRAS* mutation frequency in relapsed ALL was ∼1.9 folds higher than that of newly diagnosed ALL (27.8 vs. 14.7%; Figure 1B). In the PCGP cohort (McLeod et al., 2021), the mutation frequency of *KRAS*, *NRAS*, and *HRAS* were 13.9, 13.7, and 0% in *HRAS* among diagnostic samples and 25.5, 22.6, and 0% in relapsed samples (Supplementary Figure S3). To demonstrate the difference between B-ALL and T-cell ALL (T-ALL) as confirmed by flow cytometric immunophenotyping assay, we identified a higher *RAS* mutation frequency in newly diagnostic B-ALL patients than that in T-ALL patients (14.7 vs 0% in *KRAS*; 9.3 vs 6.7% in *NRAS*; 0.6 vs 0% in *HRAS*, Figure 1C). Because NRAS mutations were associated with B-ALL relapse, we then focused on exploring the *NRAS* mutation profiles in our study cohort. As shown in Figure 1D, most *NRAS* mutations located at G12, G13, and Q61 residues, with 52.5, 37.5, and 7.5% frequency, respectively. The *NRAS* mutations on other residues (G60, Y64, and A146) were very rare, which was in line with previous reports (Supplementary Figure S5A) (Prior et al., 2012). Similar pattern was also observed in *KRAS* mutations but not in HRAS mutations (Supplementary Figures S5B,C). To address the *NRAS* mutation profile, we retrieved the NRAS mutation data from pediatric Cancer Genome Project (PCGP) (McLeod et al., 2021) and identified a very similar pattern between our study cohort and PCGP study cohort. (upper panel, GWCMC study cohort; lower panel, PCGP study cohort; Figure 1E) (Hunger and Mullighan, 2015). To examine the association of *RAS* family mutations and ALL outcomes, we performed the survival analysis using Cox proportional hazards regression model. As shown in Supplementary Figure S4, we did not identify a significant lower overall survival (OS) was identified in ALL patients with *RAS* mutations (Hazard ratio [HR], 2.1, 95% CI, 0.6 to 6.8, *p* = 0.23, log-rank test). Similarly, the association of *KRAS* or *NRAS* mutations and ALL survival was not statistically significant, suggesting that RAS mutations might not impair the overall survival (Supplementary Figure S4). Next, we explored the effect of *RAS* mutations on ALL relapse and observed a higher risk of relapse among patients with *RAS* mutations than those with wild-type RAS (3-year cumulative relapse incidence: 18.7 ± 9.1% vs. 3.8 ± 1.3%, *p* = 0.0021, Gray test; Supplementary Figure S4). This pattern was observed in the *KRAS* mutation subgroup (*p* = 0.0012) but not in the *NRAS*-mutation subgroup (*p* = 0.18) (Supplementary Figure S4). Meanwhile, we did not identify an association of *NRAS* mutations with the therapeutic response as reflected by the minimal residual diseases (MRD) (Supplementary Table S5).

**TABLE 1:** Characteristics of enrolled patients from CCCG-ALL-2015 cohort.

Characteristics	Primary ALL (*N* = 333)	Relapse ALL (*N* = 18)	*p* Value
Age (yrs, mean ± sd)	4.8 ± 0.15	3.9 ± 0.46	0.1
Gender (Male/Female)	205/128	10/8	0.48
FAB			
L1	59	7	0.49
L2	214	5	—
L3	60	0	—
Immunophenotype			
B-ALL	303	12	0.47
T-ALL	30	0	—
Risk			
Low risk	168	4	0.46
Intermediate risk	158	12	—
High risk	7	2	—
Liver			
<2 cm	160	12	0.19
≥ 2 cm, < 5 cm	145	5	—
≥ 5 cm	28	1	—
Spleen			
<2 cm	207	12	0.43
≥ 2 cm, < 5 cm	105	6	—
≥ 5 cm	21	0	—
Mediastinal mass			
No	326	18	0.46
Yes	7	0	—
CNSL			
No	324	15	0.45
Yes	8	3	—
WBC			
<50 × 10^9^/L	263	16	0.41
≥ 50 × 10^9^/L	170	2	—
*KRAS* mutation			
No	282	13	0.16
Yes	51	5	—
*NRAS* mutation			
No	300	16	0.868
Yes	33	2	—
*HRAS* mutation			
No	331	18	0.742
Yes	2	0	—

### The Effect of *NRAS* Mutations on Acute Lymphoblastic Leukemia Leukemogenesis

**FIGURE 2:**
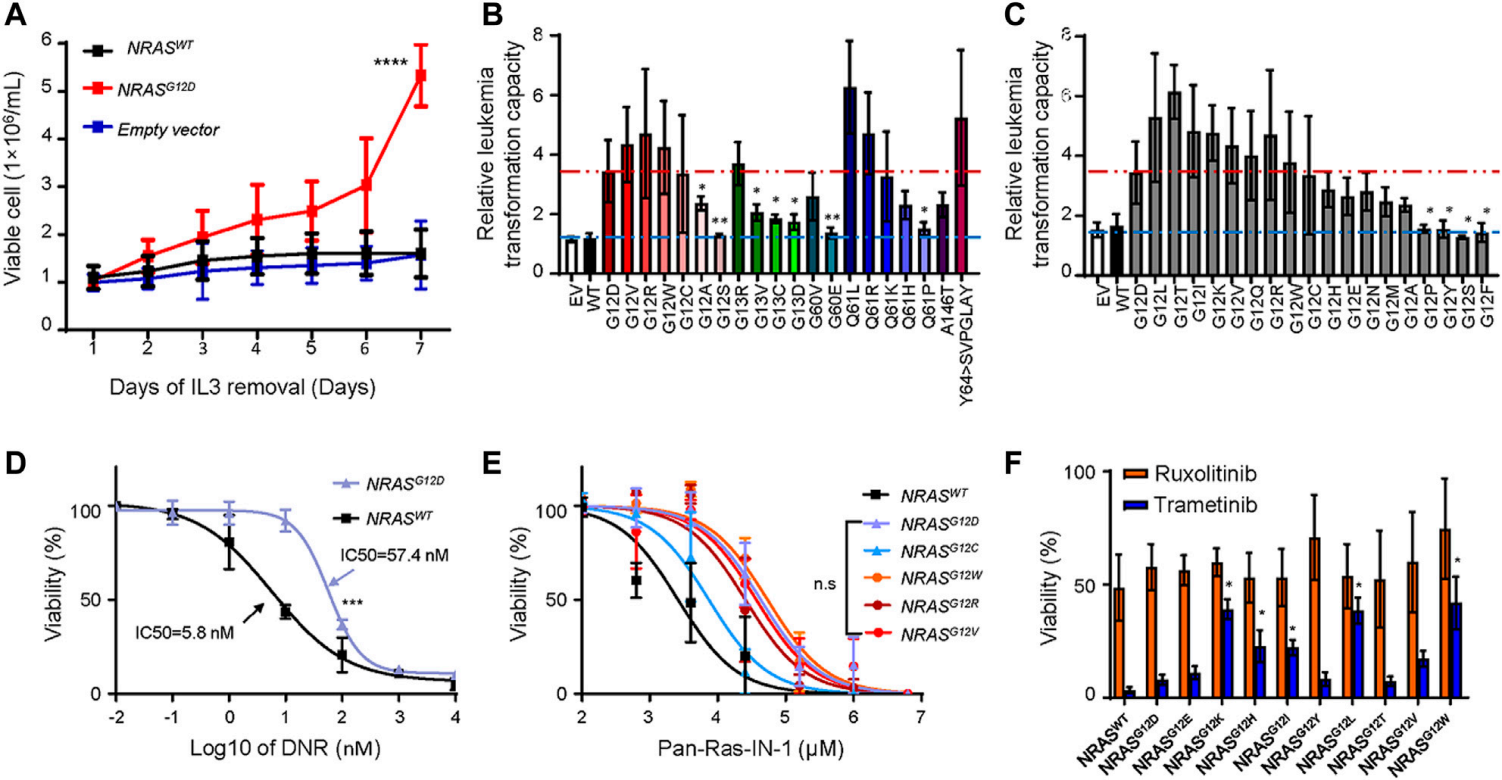
Transforming potentials and drug response of NRAS mutation s in Ba/F3 cell line. **(A)** IL-3 independent cell growth of Ba/F3 cells transduced with *NRAS* ^*G12D*^, *NRAS* ^*WT*^ and empty vector. **(B–C)** Relative leukemogenic capacity between reported NRAS mutations **(B)** and G12 saturated mutant forms **(C)**. Differences were calculated between *NRAS* ^*G12D*^ and other *NRAS* mutants by student *t* test. Dark red dash line represents the *NRAS* ^*G12D*^ transforming capacity as positive control, and light blue dash line represents non-transforming capacity. The relative capacity was calculated by the ratio of viable cells between day7 and day1. Cytotoxicity of DNR **(D)**, Pan-Ras-IN-1 **(E)**, trametinib and ruxolotinib **(F)** were examined in Ba/F3 cells with *NRAS* mutations (black line, *NRAS* ^*WT*^; purple line, *NRAS* ^*G12D*^; blue line, *NRAS* ^*G12C*^; orange line, *NRAS* ^*G12w*^; brown line, *NRAS* ^*G12R*^; red line, *NRAS* ^*G12V*^). The cell viability was measured after 72 h of drug exposure using an CCK-8 assay. All these experiments were performed in triplicate and independently repeated three times. Two-way ANOVA method was used to perform the statistical analysis for **(D,E)**, and student *t* test was applied for **(F)**”. *p* < 0.05 (*, <0.05; **, <0.01; ****, <0.0001) was considered statistically significant.

The association of *NRAS* mutations with ALL relapse has been well studied by several groups. Thus, we next experimentally evaluated the role of *NRAS* mutations in ALL leukemogenesis, we cloned all *NRAS* mutants as we identified in Figure 1. We utilized a mouse hematopoietic progenitor Ba/F3 cell line with an IL3-dependent cell growth feature as a study model to test the leukemic transformation capacity of different *NRAS* mutations. As shown in Figure 2A, ectopic over-expression of *NRAS* ^*G12D*^ but not wild-type *NRAS* or empty vector significantly induced Ba/F3 cells IL-3 independent growth (*p* < 0.0001). Using *NRAS* ^*G12D*^ as a positive control, we next tested the leukemic transformation capacity of all *NRAS* mutations and found that nine of twenty mutations (*NRAS* ^*G12V*^, *NRAS* ^*G12R*^, *NRAS* ^*G12W*^, *NRAS* ^*G12C*^, *NRAS* ^*G13R*^, *NRAS* ^*Q61L*^, *NRAS* ^*Q61R*^, *NRAS* ^*Q61K*^, NRAS^Y64>*SVPGLAY*^) significantly potentiated Ba/F3 cells transformation after removing IL3 from culture media, with the comparable or stronger capacity to *NRAS* ^*G12D*^ (Figure 2B). However, the other eleven *NRAS* mutant forms could not induce IL-3 independent growth. Since G12 residue is the mutation hot spot, we then used the saturated mutagenesis method to establish all nineteen G12 mutant forms and test their leukemic transformation capacity by the same strategy. Interestingly, not all *NRAS* G12 mutant forms could significantly activate or potentiate leukemogenesis (Figure 2C). Among of which, seven (36.8%) *NRAS* G12 mutants (*NRAS* ^*G12L*^, *NRAS* ^*G12T*^, *NRAS* ^*G12I*^, *NRAS* ^*G12K*^, *NRAS* ^*G12V*^, *NRAS* ^*G12Q*^, and *NRAS* ^*G12R*^) demonstrated stronger leukemogenic capacity than NRAS^G12D^, and another seven (36.8%) mutant forms (*NRAS* ^*G12W*^, *NRAS* ^*G12C*^, *NRAS* ^*G12H*^, *NRAS* ^*G12E*^, *NRAS* ^*G12N*^, *NRAS* ^*G12M*^, and *NRAS* ^*G12A*^) showed comparable to or a little bit weaker capacity. The remaining four (26.4%) *NRAS* G12 mutants (*NRAS* ^*G12P*^, *NRAS* ^*G12Y*^, *NRAS* ^*G12S*^, and *NRAS* ^*G12F*^) could not transform Ba/F3 cells at all. Taken together, our data suggest that not all *NRAS* mutants have leukemogenic potentials or pathogenic effects (Supplementary Table S8).

Building upon the findings above, we further asked how to target ALL cells with NRAS mutations. To address this question, we first tried to answer that whether NRAS mutations conferred resistance to conventional and novel agents, such as daunorubicin (DNR) and tyrosine kinase inhibitors. We treated with *NRAS* ^*mut*^ transformed Ba/F3 cells with DNR and found that NRAS^G12D^ ALL cells were more resistant to DNR than those with wild-type NRAS (IC50: 57.4 vs. 5.8 nM) (Figure 2D), which was in line with previous reports (Irving et al., 2014; Irving et al., 2016). Using a similar approach, we compared the effect of RAS inhibitors on *NRAS* ^*G12*^-transfected Ba/F3 cells. Ba/F3 cells transfected with distinct *NRAS* mutants (*NRAS* ^*G12D*^, *NRAS* ^*G12C*^, *NRAS* ^*G12W*^, *NRAS* ^*G12R*^, and *NRAS* ^*G12V*^) were more resistant to Pan-Ras-IN-1 (a pan-Ras inhibitor) variably than those with *NRAS* wild-type (Figure 2E). Similar results were observed for other RAS inhibitors, including Fendiline, ARS1620, and AMG510 (Supplementary Figure S6). As reported by Kirchberger et al. that MEK inhibition chemo-sensitized NRAS^G12D^-mutated ALL cells to conventional therapeutic agents (i.e., DNR and dexamethasone) (Irving et al., 2016), we thus tested the MEK inhibition response among those *NRAS* ^*G12*^ mutants transformed Ba/F3 cells. Interestingly, we identified that Ba/F3 cells with *NRAS* ^*G12E*^, *NRAS* ^*G12T*^, and *NRAS* ^*G12Y*^ mutation were as sensitive as Ba/F3 cells with *NRAS* ^*G12D*^ mutation to trametinib treatment. Meanwhile, Ba/F3 cells with *NRAS* ^*G12K*^, *NRAS* ^*G12H*^, *NRAS* ^*G12I*^, *NRAS* ^*G12L*^, *NRAS* ^*G12V*^, and *NRAS* ^*G12W*^ mutation just demonstrated a moderate response to trametinib treatment (Figure 2F). However, all tested *NRAS* ^*mut*^ transformed Ba/F3 cells did not respond to ruxolitinib, a JAK2 inhibitor.

### Translational Potential of Differential *NRAS* Mutations on Acute Lymphoblastic Leukemia Therapeutics

**FIGURE 3:**
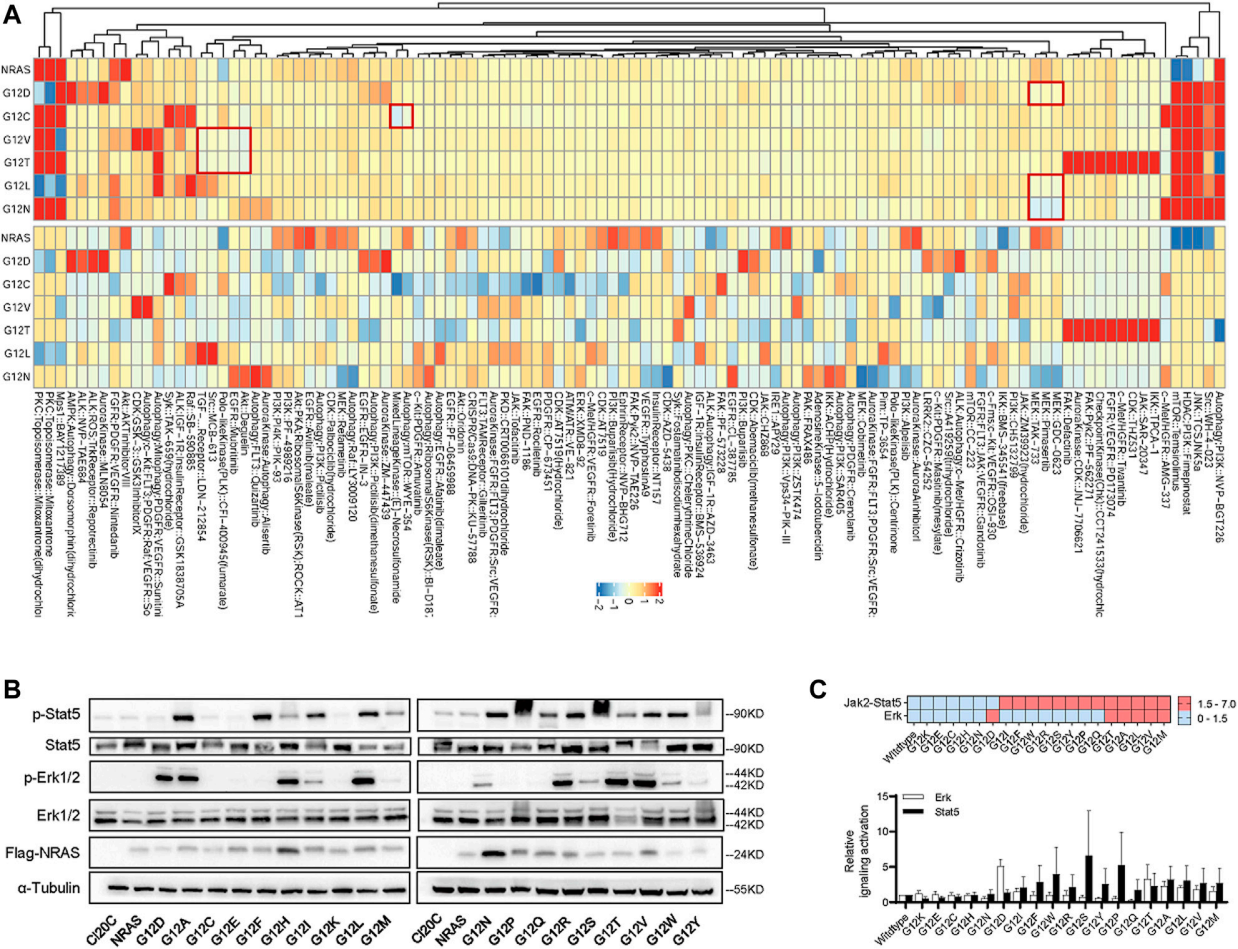
The impact of NRAS^G12^ mutation on drug sensitivity. **(A)** Normalized heatmap of HDS results (log10 of IC50 concentration) among Ba/F3 cells with different *NRAS* mutation. All these experiments were performed in triplicate and independently repeated three times. **(B)** Western blotting assay of Ba/F3 cells transduced with *NRAS* ^*G12*^ mutants. **(C)** Heatmap (upper panel) and grey intensity plot (lower panel) to Erk and Jak2-Stat5 signaling pathways activation in Ba/F3 transduced with *NRAS* ^*G12*^ mutants.

The findings above suggested that ALL cells with NRAS mutation might differently respond to signaling inhibition. To address this question, we applied HDS strategy as an attempt to identify candidate agents that could preferentially target NRAS mutations. Among the 843 tested agents (Figure 3A; Supplementary Table S9), we observed that *NRAS* ^*G12D*^ mutation well responded to MEK inhibition (GDC-0623, pimasertib, and TAK-733), which was in consistent with current clinical reports (Nakamura et al., 2013; Johnson et al., 2014; Kirchberger et al., 2018). Interestingly, *NRAS* ^*G12L*^ and *NRAS* ^*G12N*^ mutations also well responded to MEK inhibition. However, *NRAS* ^*G12C*^ mutation well responded to autophagy inhibition and mTOR inhibition (WYE−354), and mix-lineage kinase inhibition (E−Necrosulfonamide), while *NRAS* ^*G12V*^ and *NRAS* ^*G12T*^ mutations responded to Akt inhibition (deguelin), EGFR inhibitor (mubritinib), Polo−like Kinase (PLK) inhibition (CFI−400945), Src inhibition (MCB−613), and TGF−*β* receptor inhibitor (LDN−212854). The distinctive drug response among *NRAS* mutations drove us mechanistically validate our findings above. We first utilized immunoblot assay to profile the impact of NRAS mutations on Erk, Jak2-Stat5 signaling pathway. As illustrated in Figures 3B,C, we found that NRAS^G12C^, NRAS^G12K^ NRAS^G12E^, NRAS^G12H^ and NRAS^G12N^ mutations did not activate Jak2-Stat5, or Erk signaling. NRAS^G12D^ strongly activated Erk signaling, while NRAS^G12I^, NRAS^G12F^, NRAS^G12W^ and NRAS^G12R^, NRAS^G12S^, NRAS^G12Y^, NRAS^G12P^, and NRAS^G12Q^ activated Jak2-Stat5 alone. Intriguingly, NRAS^G12T^, NRAS^G12A^, NRAS^G12L^, NRAS^G12V^, and NRAS^G12M^ co-stimulated Erk and Jak2-Stat5 signaling.

## Discussion

This study identified a group of *RAS* gene mutations with a high frequency in childhood ALL. Our data analysis showed that *NRAS*, *KRAS*, and *HRAS* mutations were almost mutually exclusive within our study cohort, with only eleven patients with *KRAS* and *NRAS* mutations concurrently. In consistent with reports from several other groups (Case et al., 2008; Davidsson et al., 2010; Irving et al., 2014; Oshima et al., 2016), we did not detect any changes in the frequency of *RAS* mutations based on gender or age. Irving et al. have identified that *NRAS* mutations were associated with an increased risk of progression within hyperdiploidy standard-risk patient group by analyzing cytogenetic data from 427 children with relapsed B-ALL (Irving et al., 2016). The impact of *NRAS* mutations on childhood ALL relapse in our study cohort was slightly different from other groups (Ma et al., 2015; Irving et al., 2016), which might be explained by several factors, including but not limited to patient demographics, socioeconomic status, clinical characteristics, and study sample size. Meanwhile, we found that the detectable genomic alteration in this cohort was only 36.55% (Supplementary Figure S1), suggesting whole transcriptome sequencing is highly needed to capture all genomic lesions.

Several reports have successfully linked genetic defects (i.e., RAS pathway alterations, drug-metabolism related genes [*FPGS*, *NT5C2*, *NR3C1*, and *PRPS1*], transcription factor [*TP53*, *IKZF1*, *CREBBP*]) with ALL relapse (Mullighan et al., 2011; Tzoneva et al., 2013; Mar et al., 2014; Song et al., 2020). Many study groups have reported that RAS mutations could be detected in ∼50% relapsed ALL patients (Irving et al., 2014; Malinowska-Ozdowy et al., 2015; Oshima et al., 2016; Tasian and Hunger, 2017; Ding, 2018; Jerchel et al., 2018), indicating the importance of RAS mutations in ALL relapse. Attractively, a recent study by Zhang et al. has shown that more than 50% of relapsed pediatric ALL patients have RAS pathway mutations (*KRAS*, *NRAS*, *NF1*, *EPOR*), further consolidating the role of *RAS* mutations in relapsed ALL. In this study, we included 333 newly-diagnosed and 18 relapsed B-ALL patients, which is the largest single institutional cohort in China to systemically explore the role of *RAS* mutations in childhood ALL. The prevalence of *RAS* mutations was 25.2 and 38.9% in newly-diagnosed and relapsed children with ALL in our study cohort, respectively (Figure 1), which was in line with recent reports (Irving et al., 2014; Ma et al., 2015). It has been reported that *RAS* mutations were more likely to be enriched in high-risk ALL group, including patients with early relapse (*H-*, *K-*, and *N- RAS* mutations) and with central nervous system (CNS) involvement (*NRAS* and *KRAS* mutations) (Reshmi et al., 2017; Takashima et al., 2018). Our report here demonstrated a correlation between *KRAS* mutations and ALL relapse (Supplementary Figure S4). However, we did not observe a significant association between *RAS* mutations and treatment outcome (i.e., early treatment response (defined by MRD), OS, CNS involvement, risk stratification). There may be several possibilities, including relatively small sample size, patient demographics, and treatment protocols. Additional studies or multi-institutional cooperation were warranted to further define their relationship.

It was reported that RAS activation in malignant hematopoietic cells induces multi-drug resistance (i.e., glucocorticoids and anthracyclines) in ALL therapy (McCubrey et al., 2007; Garza et al., 2009; Irving et al., 2014). Thus, it is critical to rescue the therapeutic response so as to improve the treatment outcome. Interestingly, we found that *NRAS* mutants differed in their ability to leukemic transformation, strongly indicating that not all *NRAS* mutations are driver mutations which can be potentially targeted (Figure 2). In combination with the *in vitro* cytotoxic and signaling activation results (Figures 2, 3), we believed that those leukemogenic *NRAS* mutants might contribute to leukemogenesis and therapeutic targeting via different mechanisms, which is supported by other groups. For example, Jerchel et al. have identified that NRAS mutation-related BCP-ALL may not activate the MAPK pathway (Jerchel et al., 2018). In contrast, Chan et al. have demonstrated NRAS mutation may promote B-cell leukemogenesis via STAT5 or MAPK (Chan et al., 2020), suggesting complicated mechanisms underlying the *NRAS* mutations in B-ALL. In this study, we confirmed the well response of NRAS^G12D^ to MEK inhibition by HDS assay and western blot (Figure 3). Interestingly, we had identified a distinctive signaling activation profile. It’s well established that NRAS^G12D^ activated ERK signaling and well responded to MEK inhibition. In the meanwhile, we also found that different NRAS^G12^ mutant activated different down-steam signaling pathways (Figures 3B,C), which might partially explain the different drug response among NRAS ^G12^ mutations (Figure 3A). Though we did not find that NRAS^G12N^ activated the ERK signaling with a similar pattern as NRAS^G12D^ did, NRAS^G12N^ surprisingly well responded to ERK inhibition (Figure 3A), suggesting some compensatory mechanisms might be existed. It’s noted that NRAS^G12C^ did not activate Jak2-Stat5 or Erk signaling as shown in the western blot, and the HDS assay showed that NRAS^G12C^ was resistant to MEK or JAK inhibition, again pointing to that one targeting strategy did not fit for all NRAS^G12^ mutations. NRAS^G12T^, and NRAS^G12V^ co-stimulated Erk and Jak2-Stat5 signaling, and demonstrated a similar drug responding pattern to Akt inhibition, autophagy inhibition, and TGF-*β* inhibition. Taken together, introducing proper NRAS targeting agents into current chemotherapy regimens might be of help in further improving current ALL treatment.

## Data Availability

The datasets presented in this study can be found in online repositories. The name of the repository and accession number can be found below: National Genomics Data Center (NGDC) Genome Sequence Archive for Human (GSA-Human), https://ngdc.cncb.ac.cn/gsa-human, HRA000708
