# Supplementary material for: Effects of NRAS Mutations on Leukemogenesis and Targeting of Children With Acute Lymphoblastic Leukemia
Source: Front Cell Dev Biol. 2022 Feb 8;10:712484. doi: 10.3389/fcell.2022.712484 (PMC8861515; doi:10.3389/fcell.2022.712484)

Supplementary Figure 1

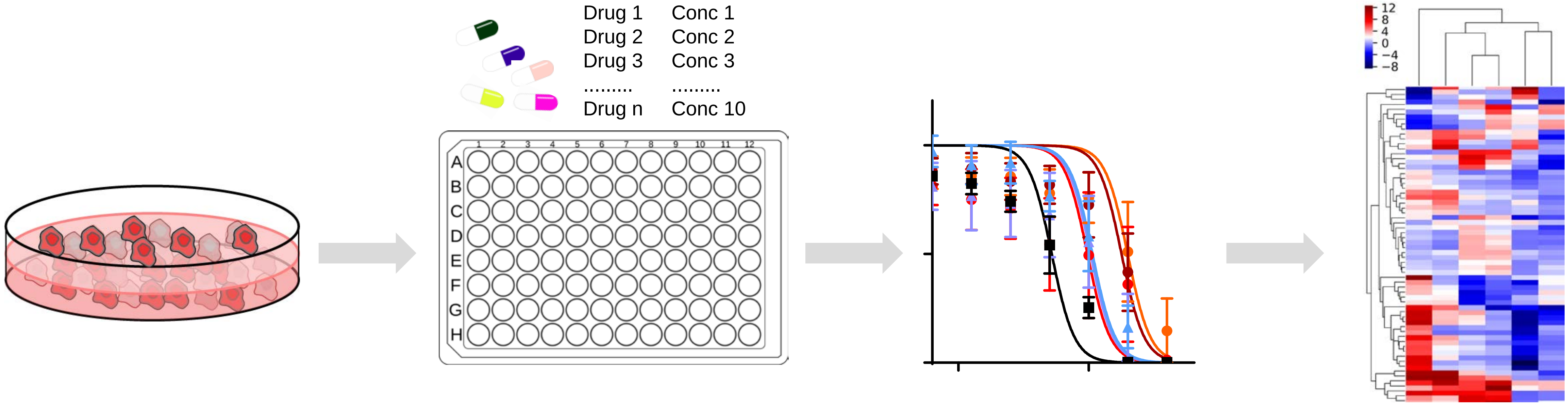

Supplementary Figure 2

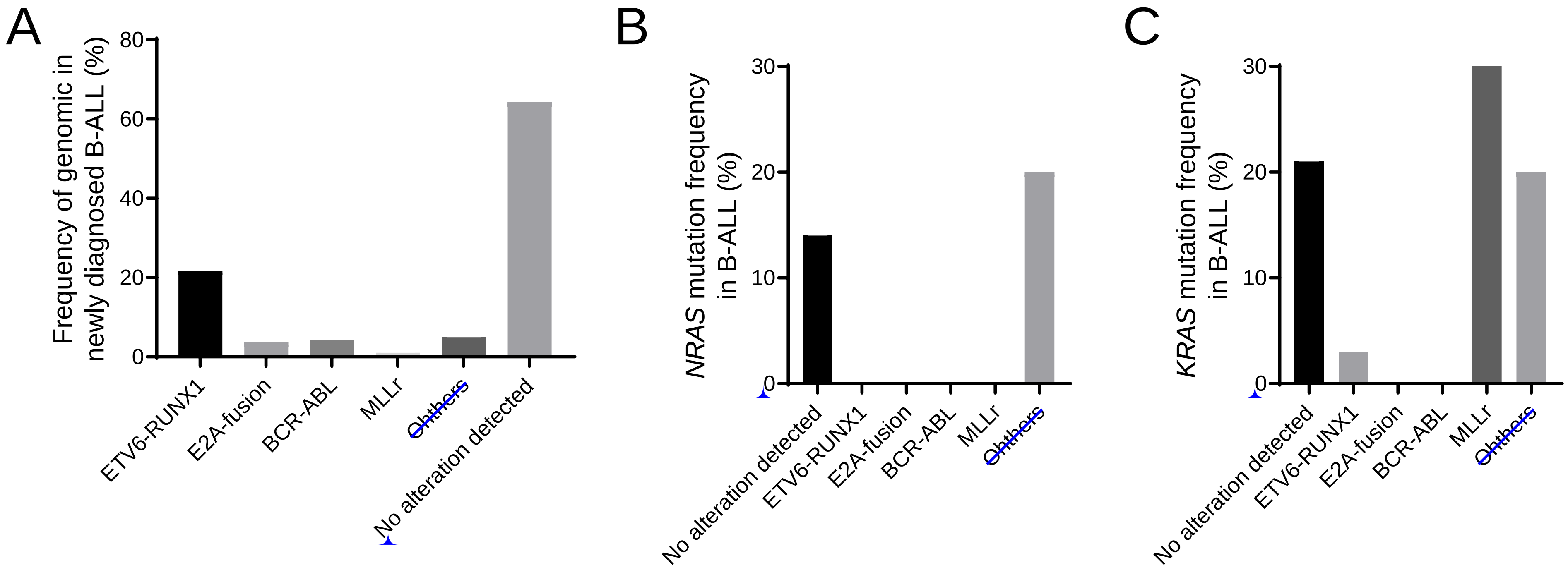

Supplementary Figure 3

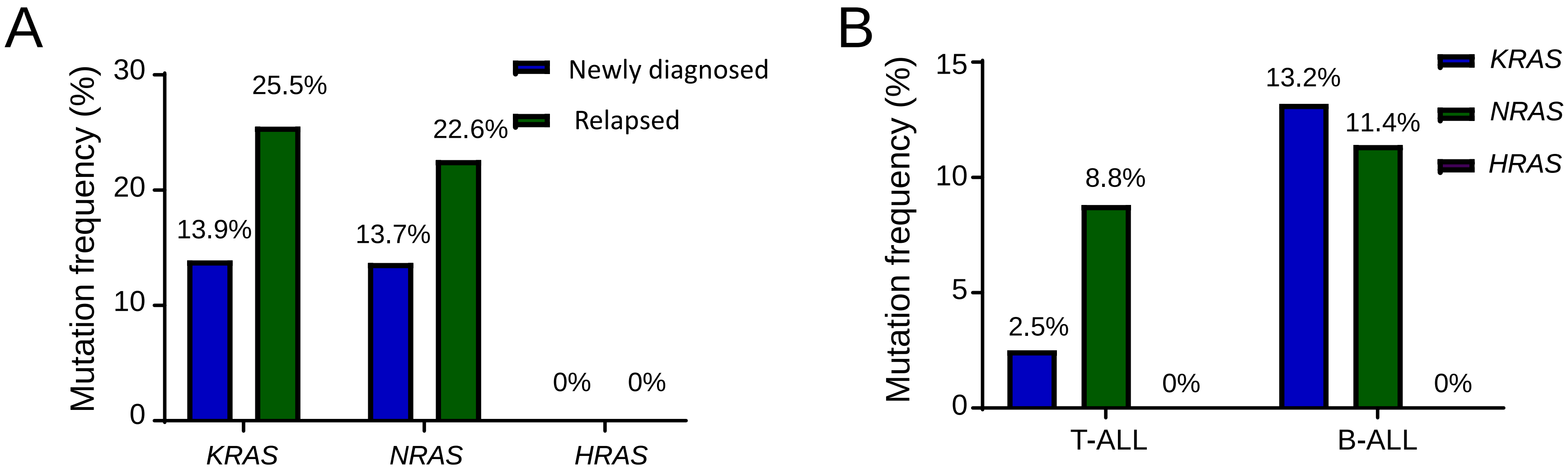

Supplementary Figure 4

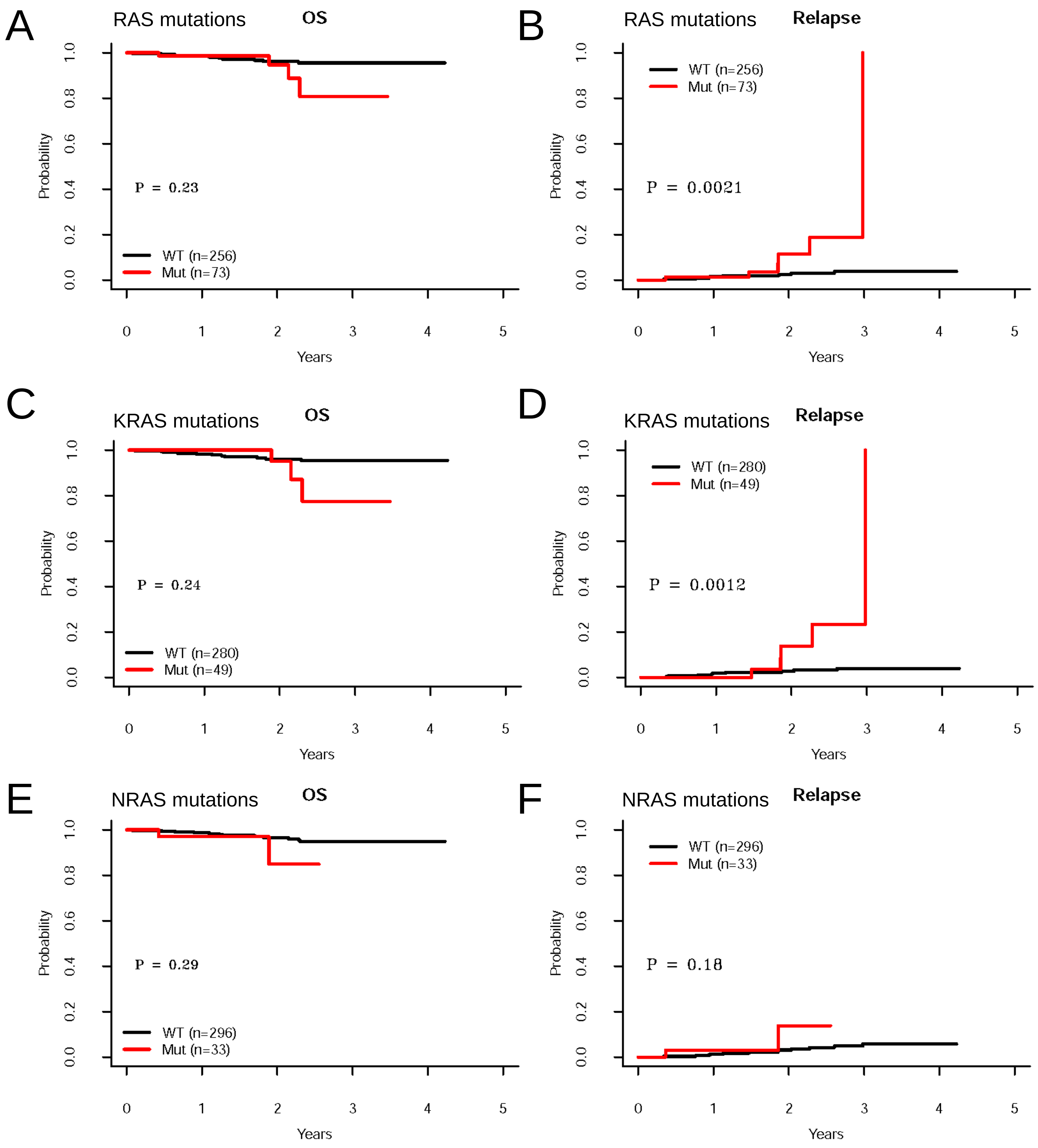

Supplementary Figure 5

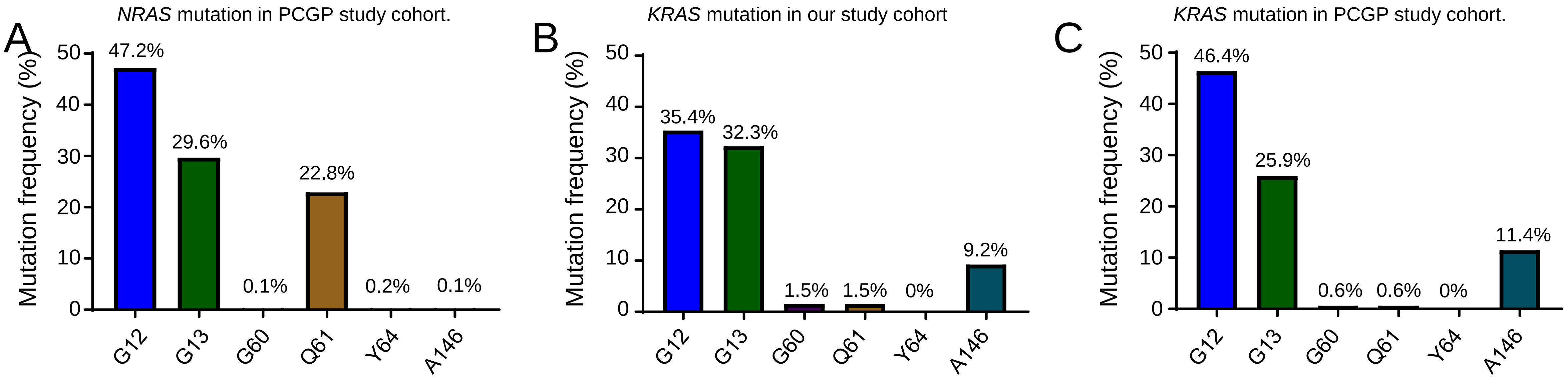

Supplementary Figure 6

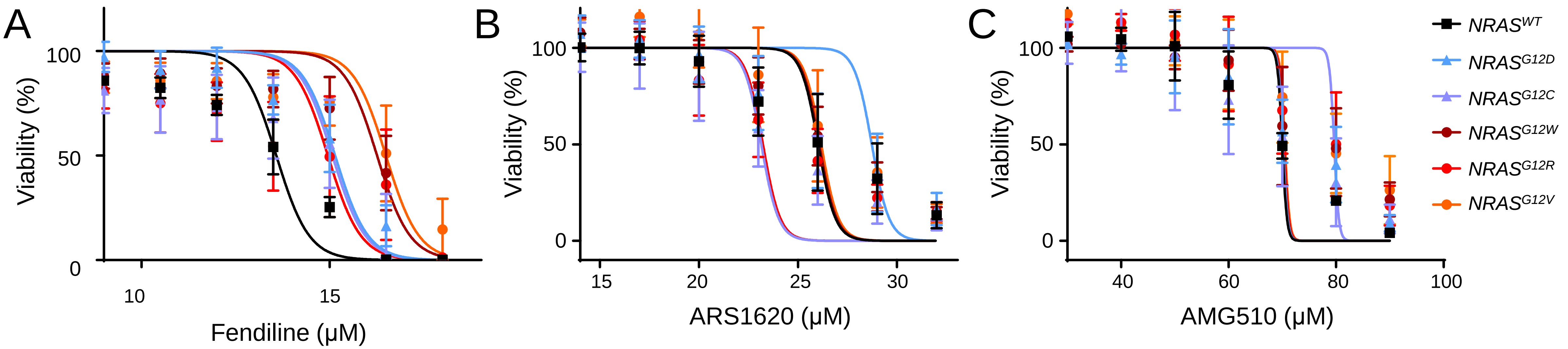

Supplement: Supplementary file 1 [file DataSheet1.pdf]
